# Physiological Candles

**Published:** 1889-12-14

**Authors:** 


### The Doctor's Pulpit.

### PHYSIOLOGICAL CANDLES.

### For Self: For Man.

"Heaven doth with us as we with torches do—not light them for hemselves."

"Spirits are not finely touched but to fine issues."

What a wonderful study is a human baby! He is purely, absolutely, and entirely selfish. If the least pain seizes him, he screams with his utmost lung-power, and wakes the whole house with his shrill and unmusical protests. What cares he that his mother has been awake for the last three nights, and is "dying" for want of sleep? How much does he concern himself though his father is an insomniac, and, if once disturbed, sleeps no more that night? The little man is himself unhappy, and is resolved that the whole world shall know it. Blessed little rascal, in that he can give himself up to utter selfishness without a blush and with no single twinge of remorse! Is he hungry? Then food he must and will have, and that at once, or woe be to the peace of the household! How happy he is, too, when he has accomplished his selfish little ends! The assurance of complete content and self-satisfaction that beams from his eyes when he looks up victoriously into his mother's face after a "screaming fight" is truly sublime. He seems to pat her metaphorically on the back with the effusiveness of an unconscious Pharisee, as if he would say, "Now you have done exactly what I wished, and what it was your duty to do; and you are a very good woman. I condescend to be pleased with you."

Such is the human baby. In him the absence or non-development of intelligence is equivalent to destitution of morals. For self, the baby is. But this is felt to be perfectly natural. All the world—that is, the baby's world—conspires with the mother to furnish him with everything he wants, or that can by any possibility be good for him; and all the reward the world asks is that he shall be "happy when he gets it."

As it is with the baby so is it with a newly-planted fruit tree. It spreads its roots in all directions, taking what it can from the soil, and stretches out its leafy branches to the sky, drinking in the rain and dew, and sunning itself in the summer air. But all for itself. For years it makes no attempt at bearing fruit. It is content to live, and thrive, and grow, and nothing else.

Now leave the baby and the young fruit tree for twenty years, and then visit them again. Is it possible to believe that this grown man is the infant of a score of years ago? He began by being exclusively, though unconsciously, selfish. What is he now? He may be selfish still; but at least he is conscious of his selfishness, and if his training have been at all intelligent is ashamed of it. To that extent, therefore—namely, the extent of having acquired the conscious power of recognising his own selfishness—he is a totally different personality from his infantile self.

But, at any rate, whatever his disposition may be, nature and the world demand and insist that the big boy or the young man shall do something other than merely eat, sleep, grow, and exact attention from all and sundry, whereas in his babyhood his mother and the whole home circle conspired to minister, without question, to his every need. Now, at the commencement of manhood his whole circle unites to press and force upon him some line of action and duty which shall demand and secure his utmost powers on the great world's behalf. The young man has been produced. He has had his day of more or less unconscious and involuntary vegetation. The probability is that it has been a happy day. He has lived for a longer or shorter period for himself alone. The time has now arrived when in the natural course of things he must play his part in the world and live for man. This is not merely the moral and commonly accepted aspect of the case; it is what physiology, too, demands and insists upon. That science declares in favour of the selfishness of the baby; but it also declares, and with equal emphasis, in favour of the unselfishness of the grown man. That which we have called selfishness in the child, partly in jest and partly for want of a better word, is the necessary preliminary and preparation for the unselfishness of the man. The baby must do what it can. It can eat, and sleep, and grow, and proclaim its wants and woes to the home world. That is all that is required of it, either by physiology or the natural instinct of its guardians. Similarly, the grown man must do what he can? And herein lies the whole point of this brief discourse. We wish to make clear, by means of the light of a physiological candle, what is the general line of service which humanity has a scientific right to require of each separate man and woman that is born into the world.

In what may be called a non-artificial state of society, Nature invites every man and woman to use his muscles and his brains. "Nature invites," we say, because she makes the use of the muscles and brains a pleasure to healthy creatures. But though her look is naturally mild, it is not the mildness of weakness, but of conscious and unlimited strength. She knows that if her invitations fail she has the rods of hunger, of nakedness, of personal shame, and of social degradation in readiness, and therefore she can well afford to be mild. Physiological Nature, though she invites every man to work, and to work for the world, does not ask him to work without profit to himself. It is a striking fact in natural economy that the more and the better work a man does for the world the more and the better is the profit to himself. This is no doubt a commonplace, but it is a commonplace that will never grow old any more than the brain or the liver will grow old; and it is a commonplace that requires to be kept in constant appreciation and healthy activity, exactly as the brain and the liver require to be kept in healthy activity.

To take one of the most familiar of all everyday examples: Nature requires the ground to be cultivated in order that man may be fed. Is it not as clear as noonday that the man who cultivates his ground most thoroughly, most intelligently, most willingly, and most cheerfully, not only renders the greatest service to his fellow-men, but brings back the largest external profit to himself, and the greatest internal profit as well in the shape of health, sleep, appetite, and pleasure? A well-known minister of religion once wrote a book with the title "Is it possible to make the best of both worlds?" He was much sneered at by very many people as a cold and calculating creature, who wanted in every condition of life to always be eating plums and drinking champagne. But most certainly, from a truly scientific point of view, which is the truly religious point of view when properly understood, the question which gave the title to the book is emphatically a wise question, and one which can only be properly answered in the affirmative. Not only is it possible to make the best of both worlds, but no man understands, at any rate, the Christian religion, or the true bearing of science either, who does not consciously, steadfastly, and always do his very utmost to "make the best of both worlds." We are not commending the doing of a man's best from the selfish motive of gaining the most. He whose chief motive power is gain of any kind to himself, is never able to do his best; and can, therefore, never secure the highest results even of which his own intelligence and powers are capable. Without any doubt whatever, science shows this, that he who yields his conscience and his intelligence to the service of the world under the guidance, approval, and mastership of an intelligence and a power external to himself, which all civilised men have agreed to call "God," renders both to the world and to himself the very best service of all kinds of which his whole man is capable. It is profoundly instructive, and inspiring beyond the power of language, to feel absolutely convinced and assured that between a reasonable religion and an intelligent science the accord is universal, perfect, and complete.

